# Ultrasound-Assisted Extraction and Characterization of Polyphenols from Apple Pomace, Functional Ingredients for Beef Burger Fortification

**DOI:** 10.3390/molecules27061933

**Published:** 2022-03-16

**Authors:** Luna Pollini, Francesca Blasi, Federica Ianni, Luca Grispoldi, Simone Moretti, Alessandra Di Veroli, Lina Cossignani, Beniamino Terzo Cenci-Goga

**Affiliations:** 1 Department of Pharmaceutical Sciences, University of Perugia, 06126 Perugia, Italy; luna.pollini@studenti.unipg.it (L.P.); francesca.blasi@unipg.it (F.B.); federica.ianni@unipg.it (F.I.); 2 Department of Veterinary Medicine, University of Perugia, 06126 Perugia, Italy; beniamino.cencigoga@unipg.it; 3 Department of Chemistry, Biology and Biotechnology, University of Perugia, 06123 Perugia, Italy; simone.moretti@molhorizon.it (S.M.); alessandradiveroli@gmail.com (A.D.V.); 4 Center for Perinatal and Reproductive Medicine, Santa Maria della Misericordia University Hospital, University of Perugia, 06132 Perugia, Italy

**Keywords:** apple pomace, waste, polyphenols, ultrasound-assisted extraction, antioxidant activity, beef burger, fortified meat, sensory evaluation, triangle test

## Abstract

Currently, there is an increasing interest to valorise agri-food waste containing bioactive compounds with potential health benefits. In this paper, the recovery of functional molecules from apple pomace, the most abundant by-product of the apple processing industry, was carried out by ultrasound-assisted extraction (UAE) on fresh and freeze-dried samples. UAE extract, obtained by double extraction of freeze-dried apple pomace, was subjected to chromatographic and spectrophotometric characterization. It showed good levels of total phenol content, high antioxidant activity, and interesting antioxidant compounds (quercetin derivatives, chlorogenic acid, phloridzin). Subsequently, freeze-dried apple pomace, containing 40.19% of dietary fibre, was used as a fortifying agent for beef burgers (4% and 8%). The results concerning colour and sensory analysis of the fortified products were graded even better than the control (0%). The improved fibre and phenol content, together with the neutral flavour, represent the most interesting characteristics of fortified burgers. The results confirm that UAE was a successful technique for extracting phenol compounds and that the addition of apple pomace represents a valid approach to increase the health properties and palatability of beef burgers, including for consumers who do not like meat.

## 1. Introduction

All around the world, food demand has increased along with food waste and by-product production, creating severe management problems and environmental pollution [1]. Generally, the by-products derived from fruit and vegetable processing contain valuable bioactive compounds with potential health benefits, including anti-inflammatory, antioxidant, and cardio protective effects [2]. It has also been reported that sometimes these phytochemicals are found in greater quantities in these by-products than in the related edible parts [3]. Therefore, the main goal of food industries is to recover bioactive compounds from waste and to reuse them in the development of new value-added products.

The recovery of bioactive compounds involves extraction as the key step, which is achieved through traditional methods or innovative methods [4]. Among the latter, ultrasound assisted extraction (UAE) has advantages such as less time and energy requirement, extraction at low temperature, and retention of the quality of the extract. The ultrasound high intensity sound waves cause disruption in the plant tissue through physical forces developed during acoustic cavitation and helps in the release of extractable components into the solvent in a shorter time by enhancing mass transport. Ultrasound is successfully employed for the extraction of polyphenols, carotenoids, aromas, and polysaccharides from plant matrices (whole plant and by-products) [5].

Apple (*Malus* sp.) pomace is one of the most abundant by-products of the apple juice industry. It consists of flesh, peel, stalks, and seeds derived from fruit processing [3]. The well-known health benefits of apple pomace have been already demonstrated in the literature, such as its role in the prevention of cardiovascular, metabolic and neurodegenerative diseases, some cancers and type 2 diabetes [6]. These beneficial properties are mainly due to the antioxidant activity of polyphenols, such as phenolic acids (especially chlorogenic acid), flavonoids (catechins, epicatechins), dihydrochalcone (phloridzin) and flavonols (quercetin glycosides) and also to the physicochemical and rheological properties (emulsion stability) of the dietary fibre. In fact, the total fibre intake has been related to its role in the protection against cardiovascular disease as evidenced by epidemiological data [3,6].

Currently, the primary usage of apple pomace is livestock feed, but recently, there is an increasing interest to use it as fortifying agent both in plant and animal origin foodstuffs [3]. As regards the use of vegetable waste or by-products as fortifying agents, it has been previously reported that the addition of wine grape pomace powder to beef meat in burgers improved fasting glucose and insulin resistance, plasma antioxidant levels, and oxidative damage markers [7]. More recently, the addition of grape seed extract, grape and orange pomace to beef patties has been studied, which demonstrated their natural antioxidant and antibacterial effects [8]. So far, apple pomace powder has been added to meat such as chicken patties, buffalo meat sausages, chicken sausages and goshtaba (typical meat preparation from India), in order to improve the dietary fibre intake, the rheological properties, emulsion stability, shelf-life (while avoiding nitrate due to the antioxidant and antimicrobial activities of polyphenols), and to lower the fat levels [9,10,11].

It must be taken into consideration that in the Mediterranean diet, a low intake of red meat is considered beneficial. Among red meat products, beef burgers are one of the most frequently consumed in developed countries, in particular those produced in fast food chains [12].

In this paper, a characterization of fresh and freeze-dried apple pomace extracts obtained by UAE, was carried out by spectrophotometric analysis (determination of total phenol content and antioxidant activity) and then by high performance liquid chromatography (HPLC) analyses with UV and Q-TOF-MS/MS (quadrupole time-of-flight tandem mass spectrometry) detectors. After apple pomace characterization, in order to improve the nutritional characteristics and the antioxidant properties of beef meat, a functional food using apple pomace was developed. Two percentages of this by-product (4% and 8%) were added to minced meat in order to produce fortified beef burgers. Their physical-chemical properties, colour, microbiological characteristics, sensory and textural properties were evaluated and compared with non-fortified beef burgers.

## 2. Results and Discussion

### 2.1. UAE Extraction of Polyphenols and Spectrophotometric Characterization

Apple pomace, the main by-product obtained after crushing and pressing apples for juice production, is a potential source of bioactive polyphenols. In this research, in order to evaluate the extraction efficiency of these compounds, the UAE technique was carried out both on fresh and freeze-dried apple pomace. Table 1 shows the values of extraction yield (%), total phenol content (TPC) and antioxidant activity of fresh and freeze-dried apple pomace.

It can be noted that the yield (%) value increases from 7.12% to 13.61% when the UAE extraction was carried out twice on fresh pomace, but a further increase (from 21.64% to 58.09%) can be noted when the UAE was performed twice on freeze-dried pomace. As regards TPC, values under 0.72 mg GAE (gallic acid equivalents)/g were obtained with fresh pomace, while up to 10.05 mg GAE/g dry weight (DW) when UAE was carried out twice on freeze-dried pomace.

Similar values of TPC (10.16 mg GAE/g) were reported by Sudha et al. (2007) [13] for dried apple pomace powder (150 μm) procured from the fruit juice industry. A large range of values were found by García et al. (2009) [14]. These authors reported values from 5.5 to 10.9 g GAE/kg for dried milled apple pomace (0.5 mm) obtained experimentally in the laboratory, or from 3.9 to 13.9 g GAE/kg for samples obtained from industrial pomace. Ferrentino and co-workers [15] studied the bio-recovery of antioxidants from apple pomace by supercritical fluid extraction (SFE) and reported values of TPC ranging from 2.52 mg GAE/g for fresh to 6.41 mg GAE/g for freeze dried apple pomace. They found that the TPC values increased (5.04–8.87 mg GAE/g for fresh and freeze-dried apple pomace, respectively) using ethanol (5%) as a co-solvent.

In order to evaluate the antioxidant activity of extracts, three complementary spectrophotometric in vitro assays were carried out. The ABTS (2,2′-azino-bis(3-ethylbenzothiazoline-6-sulfonic acid) diammonium salt) assay measures the ability of antioxidants to scavenge ABTS free radicals by electron donation, while the DPPH (2,2-diphenyl-1-picrylhydrazyl) assay evaluates the ability of antioxidants to scavenge the chromogen DPPH free radical. Finally, FRAP (ferric reducing antioxidant power) was used to evaluate the reducing capacity of the extracts. In this paper, the highest values of ABTS (27.22 mg TE/g DW), DPPH (10.50 mg TE/g DW), and FRAP (1.27 mg TE/g DW) were found for freeze-dried extract (FD2), in fact all ABTS, DPPH, and FRAP values of FD2 were two times higher than the other freeze-dried extract (FD1). Taking into consideration fresh pomace, the values were even lower both with only one (F1) or with two (F2) extractions. Ferrentino and colleagues [15] reported a wide range of DPPH values for SFE extracts: from 0.75 to 3.24 mg TE/g of extract for fresh and freeze-dried apple pomace, respectively. The authors observed that the DPPH values increased (1.33–5.99 mg TE/g for fresh and freeze-dried apple pomace, respectively), using ethanol (5%) as a co-solvent, confirming the results of TPC. Other authors reported 11.1–15.9 g ascorbic acid (AA)/kg for the DPPH assay, and 9.5–13.8 g AA/kg for the FRAP assay for apple pomace powder extracted with acetone: water in an ultrasonic bath [13].

In order to make a comparison between FD2 with a traditional method, extraction by maceration (MAC) on fresh and freeze-dried apple pomace were also carried out. It can be noted that the values of yield (%) were even higher for UAE in respect to MAC (58.09% vs. 48.65%). A similar trend can be observed for TPC (10.05 vs. 6.84 mg GAE/g DW) and antioxidant properties; in fact, values of 5.60, 7.36. and 0.82 mg TE/g DW were found for the MAC extract obtained by DPPH, ABTS, and FRAP assays, respectively.

Moreover, to better characterize the extracts, total monomeric anthocyanin content was also determined by spectrophotometric assay and a value of 6.09 mg/L (± 0.60) was found for freeze-dried UAE extract (FD2), a content higher than for the MAC extract (5.32 mg/L ± 0.07). As for TPC and antioxidant properties, the anthocyanin content varies strongly with apple variety, part of the fruit, and processing conditions; in fact, there were values from 14.0 to 74.0 mg/kg for freeze-dried or vacuum-dried pomace [16].

### 2.2. Characterization of Extracts by HPLC-UV and UHPLC-Q-TOF-MS/MS Analyses

To further characterize the phenol components of UAE extract (FD2), the UHPLC-Q-TOF-MS/MS analysis was carried out for the identification of the bioactives, while HPLC-UV analysis was performed in order to quantify them.

Table 2 shows the regression equations, R^2^, and the limits of detection (LOD) and limits of quantification (LOQ) obtained from HPLC-UV analysis of three phenol compounds used as reference standards. Chlorogenic acid, quercetin-3-O-glucoside, and phloridzin, with a concentration in the range 0.42–20.0 μg/mL, were tested in order to determine the reliability of the method in terms of linearity. The linear regression performed for each compound gave a good regression coefficient (R^2^ higher than 0.99965), which indicated a good linear relationship between the chromatographic response areas and concentrations for all compounds. The values of LOD and LOQ showed a good sensitivity for the analytical procedure used to determine phenol compounds in apple pomace.

The reliability of the method in terms of precision was also determined for the same three standard compounds with two different control solutions (with theoretical concentrations fixed at 5.00 and 15.00 µg/mL, respectively). For intra-day relative standard deviation (%RSD), one-day measures of four replicates (intra-day precision or repeatability) was considered, whereas for inter-day %RSD, the standard solution containing phloridzin, quercetin-3-O-glucoside, and chlorogenic acid was analysed for three consecutive days (inter-day precision or within laboratory reproducibility); it was possible to observe that both intra-day and inter-day precisions were acceptable (Table 3). The results showed that intra- and inter-day %RSD of the retention times (t_R_), peak areas (P_A_), and concentrations (C_P_) had low values (as regards intra-day precision: less than 0.64 for t_R_, less than 3.84 for P_A_, less than 3.91 for C_P_). Therefore, the developed method also had good precision for the simultaneous quantitative evaluation of chlorogenic acid, quercetin-3-O-glucoside, and phloridzin in apple pomace.

Figure S1 (Supplementary Materials) shows the HPLC-UV chromatographic profile of the FD2 sample, while Figure S2 shows the chemical structures of the identified polyphenols. They included chlorogenic acid, quercetin derivatives (rutinoside, galactoside, glucoside, xyloside, arabinopiranoside, arabinofuranoside, pentoside and rhamnoside), and phloridzin. Table 4 shows their content in FD2 extracts. Quercetin derivatives were quantified using regression equations of quercetin-3-O-glucoside. The compounds were identified using available standards, but also through retention time (t_R_), accurate mass parent ion peaks and secondary fragment ions data, obtained by UHPLC-Q-TOF-MS/MS analysis. It can be noted that the main compound in UAE extract was quercetin-3-O-galactoside (985.61 mg/kg DW), followed by quercetin-3-O-arabinofuranoside (536.75 mg/kg DW) and phloridzin (384.00 mg/kg DW), while the main phenolic acid was chlorogenic acid (35.71 mg/kg DW). The same compounds were also reported in other papers with a wide range of contents [3,14].

### 2.3. Chemical Composition of Apple Pomace 

The chemical compositions of apple pomace were the following: 1.25%, 2.70%, 5.88%, and 40.19% for ash, protein, moisture and fibre, respectively. These values are within the range reviewed by other authors [3,13]. Fibre was the major component of apple pomace: the total fibre value (40.19%) was obtained as the sum of insoluble (32.62%) and soluble (7.57%) fractions determined by an enzymatic kit procedure. Higher values of total dietary fibre (51.10%), insoluble fibre (36.50%) and soluble fibre (14.60%) were reported for apple pomace consisting of peel, stem, and seed [13]. Total dietary fibre was 26.5% for the apple pomace made up of a mixture of Ambrosia, Cortland, Gala, Honeycrisp, Red Delicious and McIntosh varieties [17], while other authors analysed three cultivars (Royal Gala, Granny Smith, and Liberty), and found values of total fibre ranging from 60.7 to 89.8 g 100/g, with the insoluble (56.5–81.6 g 100/g) higher than the soluble (4.14–14.33 g 100/g) fraction [18]. Recently, the use of apple pomace as a fortification ingredient in different types of food has been reviewed and a wide range for total dietary fibre (26.8–82.0%) and pectins (3.5–14.32%) described [3].

The apple pomace analysed in this paper showed a value of 6.15% for pectins with an esterification degree of 65.54%. Apple pomace is an important source of pectin, used as gelling agents and stabilizers in the food industry all around the world [19]. Pectin is an important bioactive fraction of water-soluble fibre, and takes part in lipid metabolism, processes of total cholesterol reduction, and lowering the risks of diabetes and cardiovascular diseases [20]. It has also been reported that low molecular weight or oligomeric pectins show antioxidant activity [21]. It must be highlighted that the recommended daily allowances for total fibre intake for men and women aged 19–50 are 38 g/day and 25 g/day, respectively. In fact, it has been recommended by the United States Department of Agriculture (USDA) that the population has to increase dietary fibre consumption in order to experience various health benefits. In fact, diets high in dietary fibre have been reported to promote gastrointestinal health and to reduce the risk for diverticular diseases and certain cancers [22].

### 2.4. Physical-Chemical Analysis of Fortified Burgers

The mean pH values at the beginning of the experiment ranged between 6.73 and 6.82 and remained almost constant during storage, reaching values between 6.60 and 6.64 after 96 h (Table 5). No statistically significant differences were observed between beef burgers added with different concentrations of apple pomace. On the contrary, other authors reported a decrease in pH, due to the acidic nature of apple pomace used in the addition to buffalo meat patties and goshtaba, respectively [9,11]. The mean activity water (a_w_) values at the beginning were 0.952 for beef burgers with 0% of apple pomace, 0.948 for beef burgers with 4% and 0.943 for beef burgers with 8%; after 96 h they were 0.928, 0.912 and 0.908, respectively. Beef burgers fortified with apple pomace had lower values than those with 0% after 24 h and the difference was statistically significant (*p* < 0.05).

### 2.5. Colour of Fortified Burgers

Results of the colorimetric analysis are reported in Table 5. Statistically significant differences were observed for the L* coordinate after 96 h when beef burgers with 0% apple pomace were brighter, for the a* coordinate after 72 h when beef burgers with 0% apple pomace showed higher values (more red) than those fortified with 8%, and after 96 h when 0% showed higher values than both of the others, and finally for the b* coordinate after 96 h when beef burgers with 0% apple pomace showed higher values (more yellow) than those fortified with 4% and 8% apple pomace. These results agree with those of other authors that used apple pomace in different meat products, such as in uncured, reduced-fat chicken sausages and in goshtaba [10,11]. Both authors reported a decrease in the L* value with the increase in the percentage enrichment of apple pomace. This pattern has been linked to the reduction of fat content due to its substitution with apple pomace [23,24]. Differences observed for the a* value (redness) can be explained by the presence in apple pomace of brown and yellow pigments such as carotenoids, xanthophyll and chlorophyll [11]. Variation in colour coordinates is relevant because colour is one of the main factors that determine consumer’s choices and because it can hide the spoilage of the meat [25,26]. 

### 2.6. Microbiological Analysis of Fortified Burgers

Results of the microbiological analyses are reported in Table 5. Statistically significant differences were observed after 72 and 96 h when the lactic acid bacteria population were higher in beef burgers fortified with apple pomace. This difference could be linked to the simple sugars present in apple pomace which can represent a good substrate for the development and growth of lactic acid bacteria. After 72 h, the number of total coliforms was lower in beef burgers fortified with 8% apple pomace. This may be related to the competition with the lactic acid bacteria populations. The trend of bacterial populations over time did not show substantial differences between the control and fortified burgers. This is a data of considerable importance as it demonstrates that the use of apple pomace does not lead to alterations of the beef burgers (which are already considered highly susceptible to microbial spoilage), such as faster spoilage and consequent reduction in the shelf life [27].

### 2.7. Triangle Test and Sensory Evaluation of Fortified Burgers

Results of the triangle test were as follow: 11 out 20 assessors gave the correct response for the 0% vs. 4% test, 18 out of 20 assessors gave the correct response for the 0% vs. 8% test and 16 out 20 assessors gave the correct response for the 4% vs. 8% test. According to the table provided by the ISO standard, the minimum number of correct responses needed to conclude that a perceptible difference exists based on a triangle test with 20 assessors is 11 with α = 0.05, 13 with α = 0.01 and 14 with α = 0.001. Therefore, it can be concluded that there is perceptible difference between beef burgers with 0% and 4% apple pomace with *p* < 0.05 and that there is perceptible difference between beef burgers with 0% and 8% apple pomace as well as with 4% and 8% with *p* < 0.001. It is interesting to notice that the number of correct responses were higher in the 0% vs. 8% test and 4% vs. 8% test rather than in the 0% vs. 4% test.

**Figure 1:**
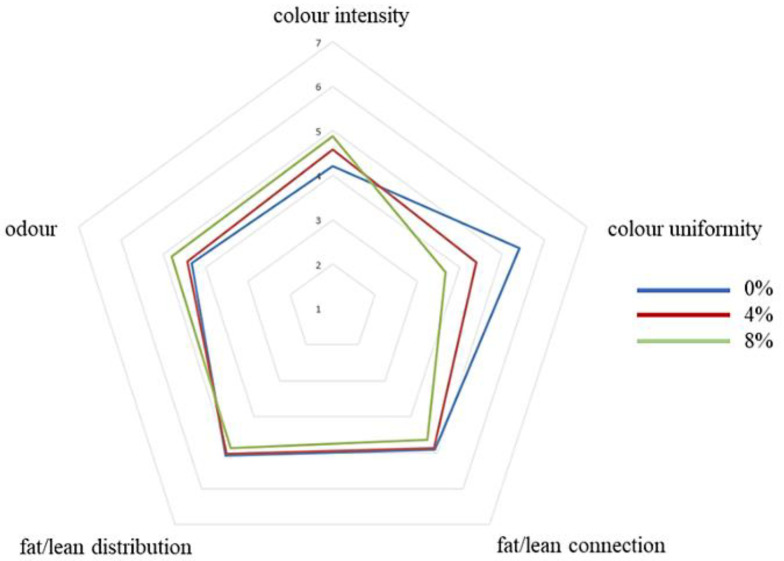
Sensory descriptive analysis. Appearance attributes and odour. (0%, control; 4%, burgers added with 4% apple pomace; 8%, burgers added with 8% apple pomace).

**Figure 2:**
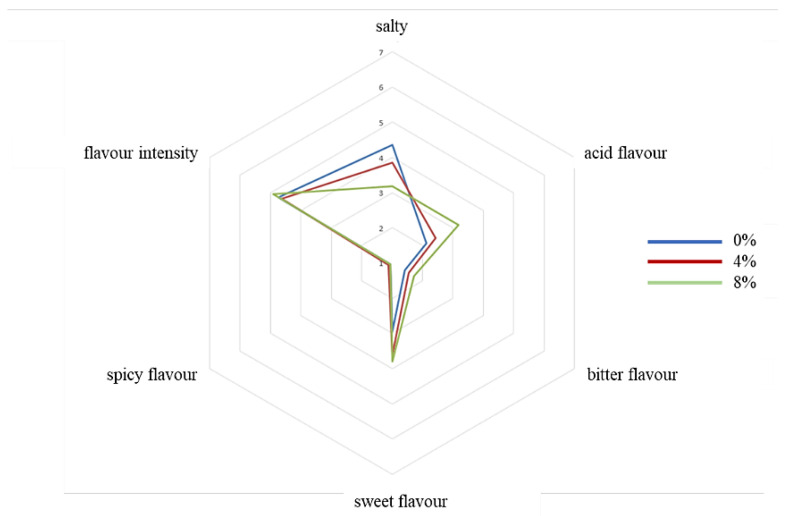
Sensory descriptive analysis. Basic tastes. (0%, control; 4%, burgers added with 4% apple pomace; 8%, burgers added with 8% apple pomace).

**Figure 3:**
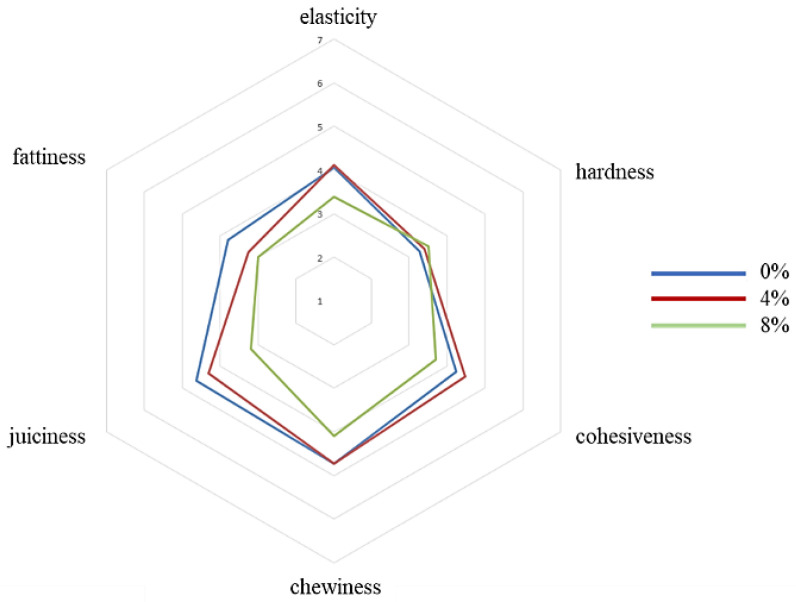
Sensory descriptive analysis. Texture attributes. (0%, control; 4%, burgers added with 4% apple pomace; 8%, burgers added with 8% apple pomace).

Results of the sensory evaluation are shown in Figure 1, Figure 2 and Figure 3.

Beef burgers with 0% apple pomace had higher colour uniformity, followed by beef burgers with 4% and lastly with 8%. On the contrary, colour intensity was higher for beef burgers with 8% apple pomace and decreased in those with 4% and 0%. Beef burgers fortified with 8% apple pomace showed higher scores for acid and sweet flavour than beef burgers with 0%. Beef burgers fortified with 8% apple pomace showed lower scores for elasticity and cohesiveness than beef burgers with 4% and 0%. Beef burgers with 0% apple pomace showed higher chewiness than those fortified with 8% apple pomace. Beef burgers fortified with 8% apple pomace showed lower scores for juiciness and fattiness than beef burgers with 0% and 4%, which were very similar. The overall acceptability was lower for beef burgers fortified with 8% apple pomace than beef burgers with 0% and 4%, which were similar. Scores from assessors that usually don’t like to consume meat showed higher overall acceptability for beef burgers fortified with 8% and 4% apple pomace. This group of assessors perceived the versions fortified with apple pomace as more uniform, less salty, less acidic, less bitter and more chewable than assessors who had declared they usually like to consume meat. Therefore, the addition of apple pomace could be a good way to make burgers more palatable to groups of consumers who usually don’t like a strong meat flavour, such as children and the elderly.

## 3. Materials and Methods

### 3.1. Materials, Reagents, Samples

Potassium chloride (KCl), sodium acetate, citric acid, and sodium hydroxide (NaOH) were from Carlo Erba Reagents (Milan, Italy). Edible-grade ascorbic acid, analysed according to Ph.Eur.9.3, was from Caelo Caesar and Loretz, GMBH (Hilden, Germany). 2,2′-azino-bis(3-ethylbenzothiazoline-6-sulphonic acid) diammonium salt (ABTS), 2,2-diphenyl-1-picrylhydrazyl (DPPH radical), 2,4,6-tripyridyl-S-triazine, sodium carbonate (Na_2_CO_3_), Folin and Ciocalteu’s phenol reagent, gallic acid, (±)-6-hydroxy-2,5,7,8-tetramethylchromane-2-carboxylic acid (Trolox), hydrochloric acid (HCl), and ethanol (EtOH) were from Sigma-Aldrich (Milan, Italy). Megazyme^®^ kit K-TDFR-100A/K-TDFR-200A 08/16 was purchased from Megazyme (Megazyme International Ireland, Bray, Ireland). Red Delicious apples and beef minced meat were purchased in a local supermarket.

### 3.2. Preparation of Apple Pomace 

Apples were washed with water and cut into pieces, separated from the seeds and petioles. Then, the pomace was obtained with a domestic fruit juice extractor (R.G.V., Como, Italy) and washed with deionized water and then freeze-dried until constant weight. Finally, it was milled with a blender (Oster, model n. 869-50R, Milwaukee, WI, USA) to obtain a homogeneous powder, maintained in an amber glass bottle at room temperature, until the successive analytical characterization. The pomace used to fortify burgers was freeze-dried.

### 3.3. Ultrasound Assisted Extraction from Fresh and Freeze-Dried Apple Pomace

The ultrasound assisted extraction was carried out according to a previous paper with slight modifications [4]. The fresh sample of apple pomace was added with EtOH:H_2_O (50:50, *v*/*v*) in a solid/liquid ratio of 1:10 (*w*/*v*) at 45 °C for 45 min in an ultrasonic bath. After centrifugation, the extracts were filtered and the solvent evaporated. An aliquot was extracted once (F1), while another aliquot was extracted twice (F2). The freeze-dried sample of apple pomace was added with EtOH:H_2_O (50:50, *v*/*v*) in a solid/liquid ratio of 1:60 (*w*/*v*) under the same extraction conditions. An aliquot was extracted once (FD1), while another aliquot was extracted twice (FD2). The yield (%) was determined as reported in another paper [28].

### 3.4. Maceration

The samples of freeze-dried apple pomace were extracted by maceration to compare the efficiency of the non-conventional extraction technique, according to Pollini et al. [27]. The powder was added with EtOH:H_2_O (50:50, *v*/*v*) in a solid/liquid ratio of 1:60 (*w*/*v*) and stirred for 4 h at room temperature. After centrifugation, the extracts were filtered and the solvent evaporated.

### 3.5. Total Phenol Content (TPC) and In Vitro Antioxidant Activity

The determination of TPC was performed according to Pagano et al. [29] with slight modifications. Folin and Ciocalteu’s phenol reagent was used and the absorbance was measured at 765 nm. The TPC was reported as mg gallic acid equivalents per gram of dry weight apple pomace (mg GAE/g DW).

The free radical-scavenging activity using ABTS assay was performed according to a previous paper [30] with slight modifications. A freshly prepared ABTS solution was added to the extracts and the absorbance was measured at 734 nm after 10 min. 

The free radical-scavenging activity of the sample was evaluated by adding the DPPH reagent (0.06 mM in ethanol). The absorbance was read after 30 min at 517 nm [30].

The reducing capacity of the extracts were evaluated using the FRAP assay according to Pollini et al. [31], with slight modifications. The apple pomace sample was added to the FRAP reagent and the absorbance was measured after 30 min at 593 nm. 

Results of ABTS, DPPH, and FRAP assays were reported as mg of Trolox equivalent per gram of dry weight pomace (mg TE/g pomace DW). Table S1 shows the calibration curve, R² and range of linearity for TPC and ABTS, DPPH, and FRAP assays.

### 3.6. Anthocyanin Content

The determination of anthocyanins was carried out according to a previous paper [29], with slight modifications. The anthocyanin content (mg/L) was calculated according to the following equation:

(1) $$\mathrm{Anthocyanin}\;\mathrm{content}\;\left(\frac{\mathrm{mg}}{L}\right)=\frac{A\times \mathrm{MW}\times \mathrm{DF}\times 1000}{\varepsilon }$$

where A = (A_520nm_ − A_700nm_)_pH = 1_ − (A_520nm_ − A_700nm_)_pH = 4.5_

MW is the molecular weight of cyanidin-3-*O*-glucoside (449.2 g/mol), DF the dilution factor and ε the molar absorptivity (26,900 L/mol × cm).

### 3.7. HPLC Analyses

#### 3.7.1. HPLC-UV Analysis

The HPLC analysis of extract was performed using a Thermo Spectra Series pump (Thermo Scientific, Rockford, IL, USA) coupled with a Shimadzu UV/Vis spectrophotometric detector SPD-10A VP UV-VIS lamp, set to a wavelength of 320 nm. The chromatographic separation was carried out with a C-18 Hypersil Gold column (250 × 4.6, 5 µm particle size). The mobile phase solvents were: (A) ACN with 0.1% of formic acid; (B) water with 0.1% of formic acid (B) For the analytical separation of the compounds, a gradient profile was employed: 0–3 min B at 100%, 5–20 min B at 85%, and 20–40 min B at 75% at a flow rate of 1 mL/min. Instrument control and data acquisition were performed using Clarity Lite software, which also allowed for signal integration. A calibration curve was built-up using chlorogenic acid, phloridzin and quercetin-3-*O*-glucoside as reference standards to quantify the phenol content of FD2 extracts. The validation of the method was performed by calculating the intra-day and inter-day, limit of detection (LOD) and limit of quantification (LOQ).

#### 3.7.2. UHPLC-Q-TOF-MS/MS Analysis

Chromatography was performed on an Agilent system (Santa Clara, CA, USA) 1290 Infinity Series High Performance Liquid Chromatography system with on-line DAD detector and coupled with Agilent Q-TOF 6540. Analytes were separated on a Kinetex XB C18 column (100 × 2.1 mm; 1.7 μm—Phenomenex, Torrance, CA, USA) and detected using both positive and negative polarity. For negative polarity analysis the mobile phases were water (A) and methanol (B) for positive polarity the mobile phases were water (A) and methanol (B) both containing 0.1% of formic acid. The gradient was the same for both polarities and initiated with 100% eluent A at 0.4 mL/min. In 33 min the eluent B increased to 50%. The gradient continued with a linear increase to 100% B in 3 min. and this condition was maintained for 4 min. The re-equilibration was 3 min for a total run time of 43 min. The column temperature was 40 °C and the injection volume was 5 μL. The DAD detector was set as primary wavelength at 254 nm with bandwidth at 4.0 nm. Slit was set at 4 nm and spectrum step was set at 2.0 nm.

The mass analyser was equipped with a heated electrospray ionization source (Dual AJS ESI). The optimized ESI temperature was set at 350 °C, the gas flow at 9 L/min., nebulizer at 35 psig, sheath gas temperature at 400 °C and sheath gas flow at 9 L/min. In positive polarity mode the capillary voltage was set 4 kV, fragmentor at 120, skimmer at 65 and octapole RF peak at 750. In negative polarity mode the capillary voltage was set 3.5 kV, nozzle voltage at 400 V, fragmentor at 120, skimmer at 65 and octapole RF peak at 750. For the spectral acquisition the AutoMS2 mode was used, setting the scan range from 100 *m*/*z* to 1300 *m*/*z*. The MS scan rate, MS/MS scan rate, isolation width MS/MS and collision energy were set at 3 spectra/s, 8 spectra/s, medium (4 u) and 15/30 respectively. The MS/MS absolute threshold was set at 50. Sheath and auxiliary gas were 40 and 15 arbitrary units, respectively. The mass extraction window was 5 ppm. Quercetin derivatives and phloridzin were analysed in positive mode, while chlorogenic acid was in negative mode.

### 3.8. Chemical Composition of Apple Pomace

The chemical composition of apple pomace was determined according to AOAC [32]. Moisture and ash contents were determined according to methods n. 925.10 and 923.03, respectively. Protein was determined as total nitrogen content (N × 6.25) using the Kjeldahl method following method n. 920.87. Total dietary fibre (TDF) and pectin were determined using the methods reported in the following 3.8.1 and 3.8.2 sections, respectively.

#### 3.8.1. Soluble and Insoluble Dietary Fibre Analysis

The procedure for soluble and insoluble dietary fibre determination was carried out using Megazyme^®^ enzymatic gravity kit, according to method n. 991.43 of the Association of Official Analytical Chemists [31]. This method included treatment with heat-stable α-amylase, protease and amylo-glucosidase. After the enzymatic treatment, the dry residue represented the insoluble dietary fibre, while the precipitate obtained with 95% EtOH represented the soluble dietary fibre.

#### 3.8.2. Pectin Extraction and Degree of Esterification

The pectin extraction was performed according to the conventional citric acid method [33] with slight modifications, while the degree of esterification (DE) of pectins was determined by the titrimetric method [34] with some modifications. The analysis was carried out as follow: 0.25 g of dried pectin was dissolved in a 250 mL Erlenmeyer flask with 1 mL of EtOH 96% and 75 mL of carbon dioxide-free water, stirring for 1 h at room temperature and sonicated in ultrasonic bath for 5 min. Then, phenolphthalein indicator was added to titrate with 0.1M NaOH until a pink coloration persisted (V_1_). Then, 10 mL of 0.5M NaOH were added to the mixture to stand for 15 min to de-esterify the pectin before 10 mL of 0.5M HCl was added, followed by shaking until the pink colour disappeared. Another three drops of phenolphthalein were added to titrate the mixture with 0.1M NaOH to a faint pink colour that persisted after vigorous shaking (V_2_). The results were reported in mg/kg of freeze-dried apple pomace, and the DE (%) was calculated as follow:

$$\mathrm{DE}\;\left(\%\right)=\frac{V_{2}}{V_{1}+V_{2}}\times 100$$

### 3.9. Production of Beef Burger Fortified with Apple Pomace 

Freeze-dried apple pomace was prepared as reported in Section 3.2, with the difference that the apple pieces were dipped in edible-grade 1% ascorbic acid solution before juice extraction. Minced meat was characterized according to AOAC [32]: moisture (70.45 ± 0.49 g/100 g meat), fats (5.68 ± 1.24 g/100 g meat), ashes (0.94 ± 0.03 g/100 g meat), and proteins (21.72 ± 0.54 g/100 g meat) were determined according to methods n. 981.10, 991.36, 950.46, and 920.153/923.03, respectively. A total of six burgers were produced in six different sessions. For each session, three batches of burgers (0%, 4%, and 8% apple pomace) were prepared. Beef burgers (100 g each) were hand prepared according to the following recipe: beef minced meat, egg (1%) and salt (2%). Freeze-dried apple pomace was incorporated in minced beef meat at 4% and 8% levels to obtain fortified burgers. Burgers without addition of apple pomace were prepared and used as control (0%). All burgers (0%, 4%, and 8%) were left overnight at 4 °C. Physical-chemical, microbiological and colour tests were carried out using raw fortified burgers (0%, 4% and 8%). Moreover, beef burgers (four of each type: 0%, 4%, and 8%) were cooked for 6 min (4 min on one side, and 2 min on the other) at 750 Watt in a domestic microwave with Tupperware MicroPro Grill equipment (Figure S3) and then they were allowed to cool down to room temperature for the successive sensory evaluation. 

### 3.10. Physical-Chemical Analysis of Fortified Burgers

To measure the pH, a S40 Seven-Multi digital pH-meter (Mettler-Toledo Italia, Novate Milanese, Italy) was used after mixing 10 g of beef burger with 90 mL of distilled water. A dew-point hygrometer HygroLab 3 (Rotronic, Huntington, NY, USA) was used to measure water activity (aw).

### 3.11. Colour Analysis of Fortified Burgers

The “ColorMeter RGB Colorimeter” app (White Marten GmbH, Stuttgart, Germany) was used to measure the colour of the samples using an iPhone XS with iOS 13.7. The methodology used has already been described by the authors in a previous manuscript [35]. Conventional colorimeters (such as the one described below) are designed to determine the colour of a single point in a uniform area. In this case we have chosen to measure the average colour of the entire product in order to replicate how the consumer perceives his portion of meat. The colour measurement of the “ColorMeter RGB Colorimeter” app was calibrated against a reference colorimeter, a Chroma Meter Minolta CR 200 (Konica Minolta Inc. Tokyo, Japan), in order to measure colour using the CIELAB scale.

### 3.12. Microbiological Analysis of Fortified Burgers

Beef burgers were analysed after their production (T0) and then after 24, 72 and 96 h (T24, T72 and T96, respectively). Samples were stored at refrigeration temperature (4 ± 2 °C) and in the dark. The methodology used for the microbiological analysis has already been described by the authors in a previous work [35]. Number of colonies were converted to the log of colony forming units per gram (log CFU/g) and the mean was calculated for each analysis. Sensitivity for spread plates was 102 CFU/g and for poured plates was 10 CFU/g, and the 95% confidence limit was set between ±37% and ±12% (i.e., plates with a number of CFU ranging from 30 to 300). Therefore, all plates with less than 30 CFU where not used for data analysis and when this applied to the lowest dilution results were recorded as <300 for the poured plate and <3000 for the spread plate [36].

### 3.13. Sensory Evaluation of Fortified Burgers

At first, a triangle test was performed according to the International Standard Organization (ISO 4120:2021) [37]. The triangle test is described as a forced-choice procedure to determine whether a perceptible sensory difference or similarity exists between samples of two products. In this study the test was used to determine if there was a perceptible difference between the three types of beef burgers (fortified with 0%, 4% and 8% apple pomace). Each pair was tested singularly (0% vs. 4%, 0% vs. 8% and 4% vs. 8%). Beef burgers were prepared and cooked as described before. Each beef burger was cut into six parts which were served in groups of three (two of the same and one different) on plastic dishes. Samples were prepared out of sight of the assessors and coded using three-digit numbers chosen randomly. Subdued illumination was used to mask any appearance differences. A total of 20 previously trained assessors were chosen and the test objectives were clearly defined. The number of assessors was calculated according to ISO 4120:2021 regulations for a test with α-risk = 0.05 (probability of concluding that a perceptible difference exists when one does not, also known as Type I error or false positive rate), β-risk = 0.1 (probability of concluding that no perceptible difference exists when one does, also known as Type II error or false negative rate) and pd = 50% (the proportion of assessments in which a perceptible difference is detected). 

A sensory evaluation was then performed. The panel consisted of the same 20 assessors. The tasters were asked to test the beef burgers for the following characteristics: colour uniformity, colour intensity, fat/lean connection, fat/lean distribution, odour (global intensity), flavour (intensity), salty flavour, acid flavour, bitter flavour, sweet flavour, spicy flavour elasticity, hardness, cohesiveness, chewiness, juiciness, fattiness, and overall acceptability. The assessors were also asked to indicate their food preferences, in particular whether they usually like to consume meat or not. Each assessor was given sheets with a 7-point (unnumbered to avoid biased assessment) scale for each characteristic: 7 = maximum intensity and 1 = minimum intensity. The evaluations were performed in individual booths, built according to the criteria of the International Standard Organization (ISO 4121:2003) [38]. Samples were prepared as described for the triangle test. Water and unsalted bread were provided to cleanse the palate between samples. Assessments were carried out under natural light at a room temperature of 20 ± 2 °C. The individual scores for each assessor were then averaged to give a score for the taste panel as a whole. Three evaluations for each different batch of beef burgers were performed. Each evaluation was carried out in different test sessions at the same time of day, between 10 and 12 a.m.

### 3.14. Statistical Analysis

Results of the chemical composition, total phenol content and antioxidant activity are reported as mean ± standard deviation (SD) of replicates. Statistical analysis was performed with StatView 5.0.1 for Mac OS (SAS Institute, Inc., Cary, NC, USA). A *t*-test comparison was performed to determine if the likelihood of observed differences between the different types of beef burgers were random. The differences were considered significant with *p* value < 0.05.

## 4. Conclusions

Apple pomace is a functional, economical, and healthy ingredient for food fortification, in particular animal origin products. In this work, the impact of UAE was evaluated in terms of recovery and profiling of bioactive compounds from apple pomace using a hydroalcoholic solvent. The results of the phenol contents and antioxidant capacity showed that UAE carried out twice, was more efficient than MAC and UAE, carried out once. The obtained results confirm that the extraction technique has a deep impact on the efficiency of the analytical procedure. These findings are relevant considering that the potential use of ultrasound extraction is promising for the extraction of antioxidants on an industrial scale. In this work, freeze-dried apple pomace was used as a meat replacement for beef burger preparation and the effect of this addition on physicochemical, textural and sensory properties was evaluated. The enrichment of red meat with apple pomace resulted in hygiene and safety characteristics comparable to non-fortified products, without leading to alterations such as fast spoilage and consequent reduction of shelf life. The outcome of this study has implications for improving the re-use of waste such as apple pomace, and in increasing the intake of fibre in the daily diet, without renouncing the beneficial effects of red meat, especially for those consumers less accustomed to the use of meat. The improved fibre content and neutral flavour are the most interesting characteristics of fortified burgers, mainly due to the addition of vegetable fibre.

## Figures and Tables

**Table 1:** Values of yield (%), total phenol content and antioxidant activity (ABTS, DPPH, and FRAP) of fresh and freeze-dried apple pomace, extracted once or twice by ultrasound-assisted extraction (mean values ± SD, *n* = 3).

Sample	Yield	TPC	ABTS	DPPH	FRAP
	%	mg GAE/g	mg TE/g	mg TE/g	mg TE/g
F1	7.12 ± 0.03	0.56 ± 0.43	0.94 ± 0.02	0.27 ± 0.02	0.05 ± 0.00
F2	13.61 ± 0.07	0.72 ± 0.11	1.23 ± 0.06	0.32 ± 0.02	0.08 ± 0.00
FD1	21.64 ± 0.21	5.49 ± 0.32	12.63 ± 0.36	5.87 ± 0.38	0.40 ± 0.00
FD2	58.09 ± 0.43	10.05 ± 0.86	27.22 ± 3.63	10.50 ± 0.22	1.27 ± 0.10
MAC	48.65 ± 1.40	6.84 ± 0.38	5.60 ± 0.32	7.36 ± 0.84	0.82 ± 0.05

F, fresh apple pomace; FD, freeze-dried apple pomace; 1, UAE one time; 2, UAE two times; MAC, maceration; TPC, total phenol content; GAE, gallic acid equivalents; ABTS, 2,2′-azino-bis(3-ethylbenzothiazoline-6-sulfonic acid) diammonium salt; DPPH, 2,2-diphenyl-1-picrylhydrazyl; FRAP, ferric reducing antioxidant power; TE, Trolox equivalents.

**Table 2:** Regression equation, R^2^, limits of detection (LOD), and limits of quantification (LOQ) of standard compounds analyzed by HPLC-UV.

Standard Compounds	Linearity Range	Regression Equation		R^2^	LOD	LOQ
	μg/mL	Slope	Intercept		μg/mL	μg/mL
Chlorogenic acid	0.42–20.0	52.38	2.28	0.99985	0.55	1.67
Quercetin-3-*O*-glucoside	0.42–20.0	10.57	4.34	0.99965	0.84	2.54
Phloridzin	0.42–20.0	10.37	2.46	0.99968	0.80	2.42

**Table 3:** Evaluation of precision (%RSD) for retention times (t_R_), peak areas (P_A_), and polyphenol concentrations (C_P_*)* at two different concentrations (5.00 and 15.00 µg/mL).

5.00 µg/mL	Intra-Day Precision (*n* = 4, Mean), %RSD									Inter-Day		
Phenolic compound	Day 1			Day 2			Day 3			(*n* = 12, mean), %RSD		
	t_R_	P_A_	C_P_	t_R_	P_A_	C_P_	t^a^	P_A_	C_P_	t_R_	P_A_	C_P_
Chlorogenic acid	0.47	0.97	0.98	0.35	2.93	2.95	0.15	2.63	2.66	0.58	2.38	2.64
Quercetin-3-*O*-glucoside	0.33	1.23	1.34	0.12	1.29	1.40	0.23	0.11	1.20	0.30	2.49	1.83
Phloridzin	0.64	1.45	1.52	0.03	1.27	1.33	0.02	0.72	0.75	0.47	2.72	4.64
**15.00 µg/mL**	**Intra-day precision (*n* = 4, mean), %RSD**									**Inter-day**		
Phenolic compound	Day 1			Day 2			Day 3			(*n* = 12, mean), %RSD		
	t_R_	P_A_	C_P_	t_R_	P_A_	C_P_	t_R_	P_A_	C_P_	t_R_	P_A_	C_P_
Chlorogenic acid	0.18	1.24	1.25	0.19	0.84	0.84	0.61	1.10	1.10	0.56	1.48	1.48
Quercetin-3-*O*-glucoside	0.13	2.76	2.84	0.16	0.95	0.97	0.14	1.65	1.70	0.27	4.82	4.96
Phloridzin	0.01	2.66	2.70	0.22	0.36	0.37	0.09	3.84	3.91	0.17	6.04	6.14

**Table 4:** Phenol content (mg/kg) of FD2 extract (mean values ± SD, *n* = 3).

n° Peak ^1^	t_R_ (Min)	Molecular Ion	Compound	mg/kg DW
1	21.66	353.0878	chlorogenic acid	35.71 ± 2.91
2	30.37	611.1471	quercetin-3-*O*-rutinoside	<LOD
3	30.98	465.1028	quercetin-3-*O*-galactoside	985.61 ± 2.81
4	31.38	465.1028	quercetin-3-*O*-glucoside	33.53 ± 3.71
5	32.48	435.0922	quercetin-3-*O*-xyloside	274.40 ± 0.46
6	33.05	435.0922	quercetin-3-*O*-arabinopiranoside	<LOD
7	33.40	435.0922	quercetin-3-*O*-arabinofuranoside	536.75 ± 8.35
8	33.74	435.0922	quercetin-3-*O*-pentoside	16.02 ± 6.90
9	34.08	449.1078	quercetin-3-*O*-rhamnoside	301.14 ± 7.61
10	36.68	437.1442	phloridzin	384.00 ± 7.90

^1^ The peak number correspond to the compounds reported in Figure S1, identified by UHPLC-Q-TOF-MS/MS in positive mode, except for chlorogenic acid; LOD, limit of detection.

**Table 5:** Results of the physical-chemical, colorimetric and microbiological analyses.

		T0			T24	
	0%	4%	8%	0%	4%	8%
pH	6.82 ± 0.01	6.78 ± 0.01	6.73 ± 0.01	6.73 ± 0.01	6.74 ± 0.01	6.79 ± 0.03
a_w_	0.95 ± 0.00	0.95 ± 0.01	0.94 ± 0.00	0.95 ± 0.00	0.94 ± 0.01	0.93 ± 0.00
*Colour coordinates*						
Lightness (L*)	23.6 ± 2.0	29.8 ± 6.3	23.4 ± 2.7	34.4 ± 13.5	23.8 ± 3.3	28.2 ± 7.4
Redness (a*)	15.2 ± 1.6	10.6 ± 1.3	8.6 ± 0.9	12.2 ± 5.9	10.0 ± 0.7	8.6 ± 0.9
Yellowness (b*)	19.0 ± 1.6	18.6 ± 2.5	16.6 ± 2.1	17.2 ± 6.3	19.0 ± 1.4	19.2 ± 1.6
PCA (log CFU/g)	5.89 ± 0.06	5.92 ± 0.06	5.93 ± 0.10	5.89 ± 0.01	5.84 ± 0.03	5.64 ± 0.01
MRS (log CFU/g)	5.78 ± 0.45	6.10 ± 0.04	6.03 ± 0.22	6.36 ± 0.13	6.31 ± 0.23	6.35 ± 0.09
M17 (log CFU/g)	6.35 ± 0.04	6.42 ± 0.03	6.29 ± 0.00	6.25 ± 0.03	6.08 ± 0.08	6.32 ± 0.10
BP (log CFU/g)	4.21 ± 0.26	4.58 ± 0.22	4.46 ± 0.29	3.72 ± 0.31	3.91 ± 0.21	3.93 ± 0.09
VRBG (log CFU/g)	4.71 ± 0.18	4.53 ± 0.09	4.56 ± 0.22	4.54 ± 0.21	4.60 ± 0.19	4.40 ± 0.27
VRBL (log CFU/g)	4.39 ± 0.32	3.80 ± 0.45	3.99 ± 0.04	4.32 ± 0.24	4.45 ± 0.31	4.41 ± 0.18
ENT (log CFU/g)	2.98 ± 0.03	3.42 ± 0.52	3.29 ± 0.20	3.11 ± 0.16	3.30 ± 0.26	3.10 ± 0.14
		**T72**			**T96**	
	**0%**	**4%**	**8%**	**0%**	**4%**	**8%**
pH	6.68 ± 0.06	6.62 ± 0.01	6.63 ± 0.01	6.64 ± 0.02	6.60 ± 0.03	6.60 ± 0.05
a_w_	0.94 ± 0.00	0.93 ± 0.00	0.93 ± 0.01	0.93 ± 0.00	0.91 ± 0.00	0.91 ± 0.00
*Colour coordinates*						
Lightness (L*)	13.2 ± 5.6	15.6 ± 4.0	19.0 ± 5.5	17.4 ± 3.0	12.6 ± 0.6	11.0 ± 1.4
Redness (a*)	6.6 ± 0.9	4.6 ± 2.0	3.6 ± 0.6	7.2 ± 0.5	4.6 ± 0.6	4.6 ± 0.6
Yellowness (b*)	8.6 ± 4.2	9.4 ± 2.5	11.2 ± 3.0	15.2 ± 1.3	10.0 ± 1.0	9.4 ± 1.5
PCA (log CFU/g)	6.59 ± 0.21	6.60 ± 0.18	6.51 ± 0.18	6.94 ± 0.23	7.25 ± 0.23	6.90 ± 0.30
MRS (log CFU/g)	6.46 ± 0.24	6.99 ± 0.21	7.04 ± 0.15	6.78 ± 0.30	7.00 ± 0.37	6.99 ± 0.31
M17 (log CFU/g)	6.84 ± 0.57	7.50 ± 0.17	7.45 ± 0.22	7.05 ± 0.18	7.58 ± 0.06	7.55 ± 0.11
BP (log CFU/g)	4.07 ± 0.24	4.17 ± 0.15	4.25 ± 0.22	3.99 ± 0.30	3.96 ± 0.24	3.86 ± 0.22
VRBG (log CFU/g)	4.79 ± 0.22	4.98 ± 0.26	4.95 ± 0.29	4.86 ± 0.66	5.12 ± 0.31	4.96 ± 0.40
VRBL (log CFU/g)	4.97 ± 0.10	5.04 ± 0.31	4.67 ± 0.28	3.75 ± 0.26	4.19 ± 0.57	3.69 ± 0.27
ENT (log CFU/g)	3.58 ± 0.11	3.26 ± 0.24	3.14 ± 0.29	3.45 ± 0.42	3.74 ± 0.34	3.38 ± 0.20

Results are expressed as means ± standard deviation (*n* = 6). 0%, control; 4%, burgers added with 4% apple pomace; 8%, burgers added with 8% apple pomace. a_w_, activity water; CFU/g, colony forming units per gram; PCA, plate count agar, total mesophilic aerobic flora; MRS, Man, Rogosa and Sharpe agar, *Lactobacillus* spp.; M17 agar, *Lactococcus* spp.; BP, Baird Parker agar, *Staphylococcus* spp.; VRBG, violet red bile glucose agar, *Enterobacteriaceae*; VRBL, violet red bile lactose agar, total coliforms; ENT, enterococcus agar, enterococci.

## Data Availability

Not applicable.

## Supplementary Materials

The following supporting information can be downloaded at https://www.mdpi.com/article/10.3390/molecules27061933/s1:, Figure S1: HPLC-UV chromatographic profile of UAE extract, from double extraction of freeze-dried apple pomace (FD2).1 chlorogenic acid; 2 rutin; 3 quercetin-3-*O*-galattoside; 4 quercetin-3-*O*-glucoside; 5 quercetin-3-*O*-xyloside; 6 quercetin-3-*O*-arabinopiranoside; 7 quercetin-3-*O*-arabinofuranoside; 8 quercetin-*O*-pentoside; 9 quercetin-3-*O*-rhamnoside; 10 phlo-ridzin; Figure S2: Chemical structures of the main quantified compounds; Table S1: Cooking equipment (a) and cooked beef burgers with different apple pomace addition: 0 % (b), 4 % (c), and 8 % (d).

## Sample Availability

Samples of the compounds are not available from the authors.
