# Supplementary material for: Ultrasound-Assisted Extraction and Characterization of Polyphenols from Apple Pomace, Functional Ingredients for Beef Burger Fortification
Source: Molecules. 2022 Mar 16;27(6):1933. doi: 10.3390/molecules27061933 (PMC8956034; doi:10.3390/molecules27061933)
Supplement: Supplementary file 1 [file molecules-27-01933-s001.zip › molecules-1639257-supplementary.pdf]

Supplementary Material

Ultrasound-Assisted Extraction and Characterization of Apple Pomace, Functional Ingredient for Beef Burger Fortification

Luna Pollini [1,†], Francesca Blasi [1,†], Federica Ianni [1], Luca Grispoldi [2,*], Simone Moretti [3], Alessandra Di Veroli [3], Lina Cossignani [1,4,*] and Beniamino Terzo Cenci-Coga [2]

[1] Department of Pharmaceutical Sciences, University of Perugia, 06126 Perugia, Italy; luna.pollini@studenti.unipg.it (L.P.); francesca.blasi@unipg.it (F.B.); federica.ianni@unipg.it (F.I.)
[2] Department of Veterinary Medicine, University of Perugia, 06126 Perugia, Italy; beniamino.cencigoga@unipg.it
[3] Department of Chemistry, Biology and Biotechnology, University of Perugia, 06123 Perugia, Italy; simone.moretti@molhorizon.it (S.M.); alessandra.diveroli@unipg.it (A.D.V.)
[4] Center for Perinatal and Reproductive Medicine, University of Perugia, Santa Maria della Misericordia University Hospital, 06132 Perugia, Italy
* Correspondence: lina.cossignani@unipg.it (L.C.); luca.grispoldi@unipg.it (L.G.); Tel.: +39-075-585-7959 (L.C.); +39-075-585-7935 (L.G.)

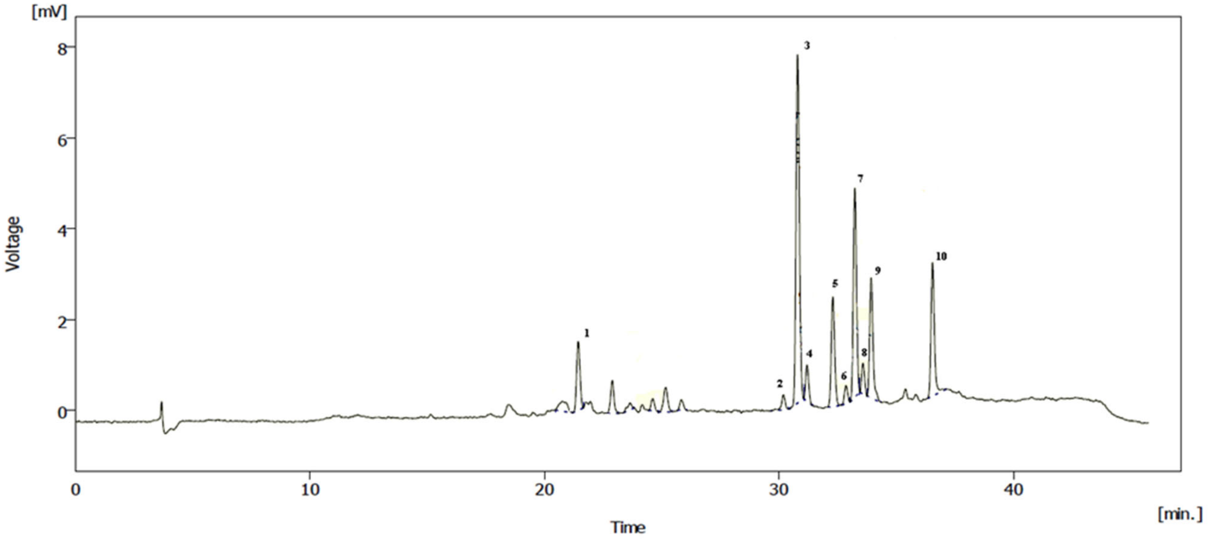

Figure S1. HPLC-UV chromatographic profile of UAE extract, from double extraction of freeze-dried apple pomace (FD2).[1] chlorogenic acid; [2] rutin; [3] quercetin-3-O-galattoside; [4] quercetin-3-O-glucoside; [5] quercetin-3-O-xyloside; [6] quercetin-3-O-arabinopiranoside; [7] quercetin-3-O-arabinofuranoside; [8] quercetin-O-pentoside; [9] quercetin-3-O-rhamnoside; [10] phloridzin.

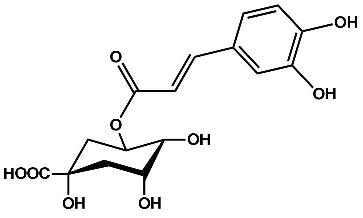

Chlorogenic acid

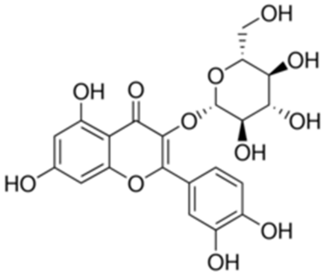

Quercetin-3-*O*-galactoside

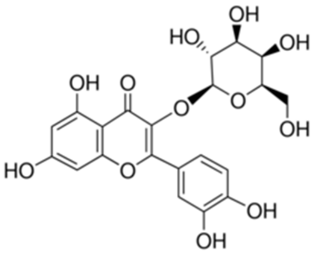

Quercetin-3-O-glucoside

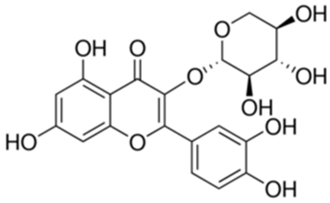

Quercetin-3-*O*-xyloside

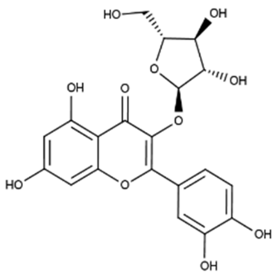

Quercetin-3-*O*-arabinofuranoside

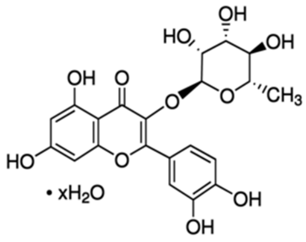

Quercetin-3-*O*-rhamnoside

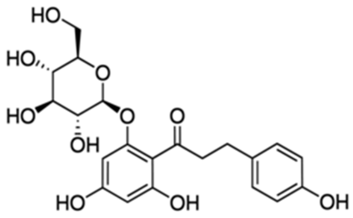

Phloridzin

Figure S2. Chemical structures of the main quantified compounds.

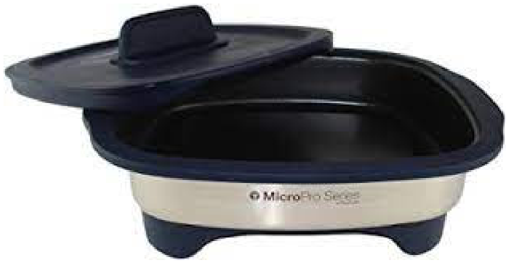

(a)

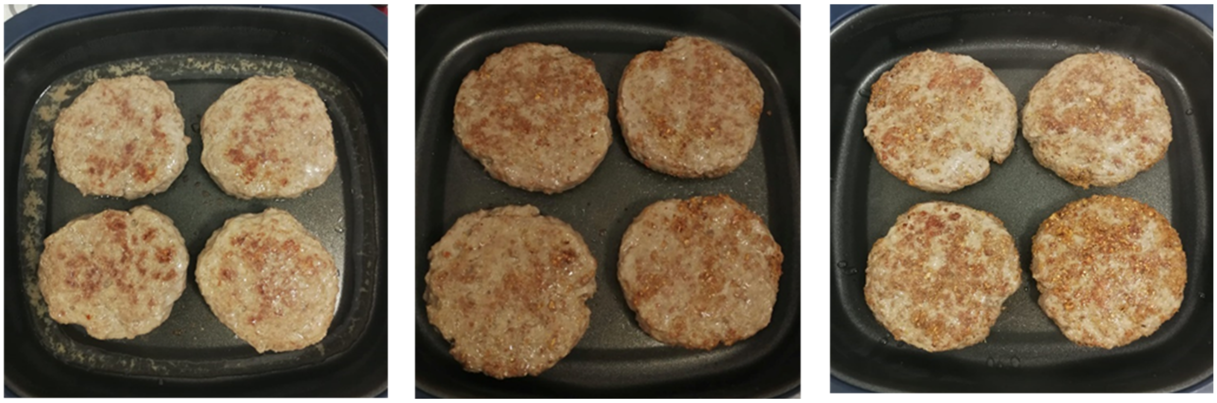

(b) **(c)** **(d)**

Figure S3. Cooking equipment (**a**) and cooked beef burgers with different apple pomace addition:.0 % (**b**), 4 % (**c**), and 8 % (**d**).

Table S1. Calibration curve, R2, and range of linearity of spectrophotometric *in vitro* assays.

	Linearity range	Calibration curve	R2
**TPC**	6.25–100 μg/mL	y = 0.0121x - 0.0262	0.9994
**ABTS**	0.01–0.3 μg/mL	y = -1.5097x + 0.6673	0.9992
**DPPH**	0.5-250.0 μg/mL	y = 1.4259x + 0.6499	0.9997
**FRAP**	0.5-10 μg/mL	y = 96.305x + 0.0368	0.9995
